# A case of laparoscopic sigmoidectomy using thermography for colonic blood flow assessment

**DOI:** 10.1186/s40792-023-01752-2

**Published:** 2023-09-25

**Authors:** Tomohiro Kako, Masahiro Kimura, Ryo Nomura, Shuhei Uehara, Hiroshi Uematsu, Seiichi Nakaya, Yuzo Maeda, Ken Tsuboi, Koshiro Harata, Shuji Takiguchi

**Affiliations:** 1 Department of Gastroenterological Surgery, Nagoya City University East Medical Center, 1-2-23 Wakamizu, Chikusa-ku, Nagoya, Aichi Japan; 2 Department of Gastroenterological Surgery, Toyota Memorial Hospital, 1-1 Heiwa-cho, Toyota, Aichi Japan; 3 Department of Gastroenterological Surgery, Nagoya City University Hospital, 1 Kawasumi, Mizuho-cho, Mizuho-ku, Nagoya, Aichi Japan; 4 Department of Gastroenterological Surgery, Nagoya City University Midori Municipal Hospital, 1-77 Shiomigaoka, Midori-ku, Nagoya, Aichi Japan

**Keywords:** Thermography, Indocyanine green, Colonic blood flow

## Abstract

**Background:**

Indocyanine green (ICG) fluorescence imaging is widely used in gastrointestinal surgery and is considered useful for reducing anastomotic leakage; however, because ICG remains in the tissue for a certain amount of time, we occasionally must re-evaluate colonic blood flow over a short time period during surgery. Herein, we verify the usefulness of thermography (TG) for evaluating colonic blood flow in a patient who underwent a laparoscopic sigmoidectomy for sigmoid colon cancer.

**Case presentation:**

The patient is 43-year-old man who underwent laparoscopic resection of the sigmoid colon for colon cancer. After vascular treatment of the colonic mesentery, ICG/TG identified the boundary between ischemic and non-ischemic colon tissues. An additional 2 cm of colonic mesentery was resected because of the presence of a diverticulum noted at the intended site of oral anastomosis when attaching the anvil head. After additional vascular treatment of the colonic mesentery and administration of ICG, fluorescence was observed in the colon; however, TG identified the zone of the temperature transition on the surface of the colonic mesentery, even after additional colonic mesentery resection in the same region as previously observed. This zone was used as the cut-off line. There were no complications, such as anastomotic leakage, after the surgery.

**Conclusion:**

Although accumulation of similar cases is necessary, TG has the potential for use as an auxiliary diagnostic tool in clinical practice. TG can depict the presence or absence of blood flow based on surface temperature without the use of imaging agents, and is inexpensive and easy to perform.

## Background

Indocyanine green (ICG) fluorescence imaging is widely used in gastrointestinal surgery and is considered useful for reducing anastomotic leakage [1]. Although the half-life is as short as 3 min [2], our experience has shown that ICG remains in the tissue for a certain period of time and re-evaluating colonic blood flow over a short time period during surgery is difficult. Thermography (TG) is a method of evaluating blood flow by measuring the temperature of the organ surface. Although TG has been used in industry and the military, it is possible to perform non-contact, non-invasive examinations using TG, and it has been reported that TG is useful in the field of medicine [3–5]. Herein, we verify the usefulness of TG for evaluating colonic blood flow in a patient who underwent a laparoscopic sigmoidectomy for sigmoid colon cancer.

## Case presentation

The patient was a 43-year-old man with a fecal specimen that was positive for occult blood during a routine check-up. He underwent a colonoscopy, which revealed a type I serrated polyp (SP) in the sigmoid colon 20 cm from the anal verge. He was referred to our hospital for an endoscopic mucosal resection (EMR). The histopathologic assessment of the resected specimen showed infiltration into the submucosal layer and he was referred to the Department of Digestive Surgery for an additional resection.

The medical history included hypertension, hyperlipidemia, and elevated hepatobiliary enzymes. He was 158 cm tall and weighed 53.2 kg. Blood tests showed elevated liver enzymes, but no other abnormalities, and tumor markers were within the normal ranges. A preoperative CT scan was negative for distant metastases and enlarged lymph nodes, and colonoscopy revealed a scar at the site of the EMR in the sigmoid colon 20 cm from the anal verge.

**Fig. 1:**
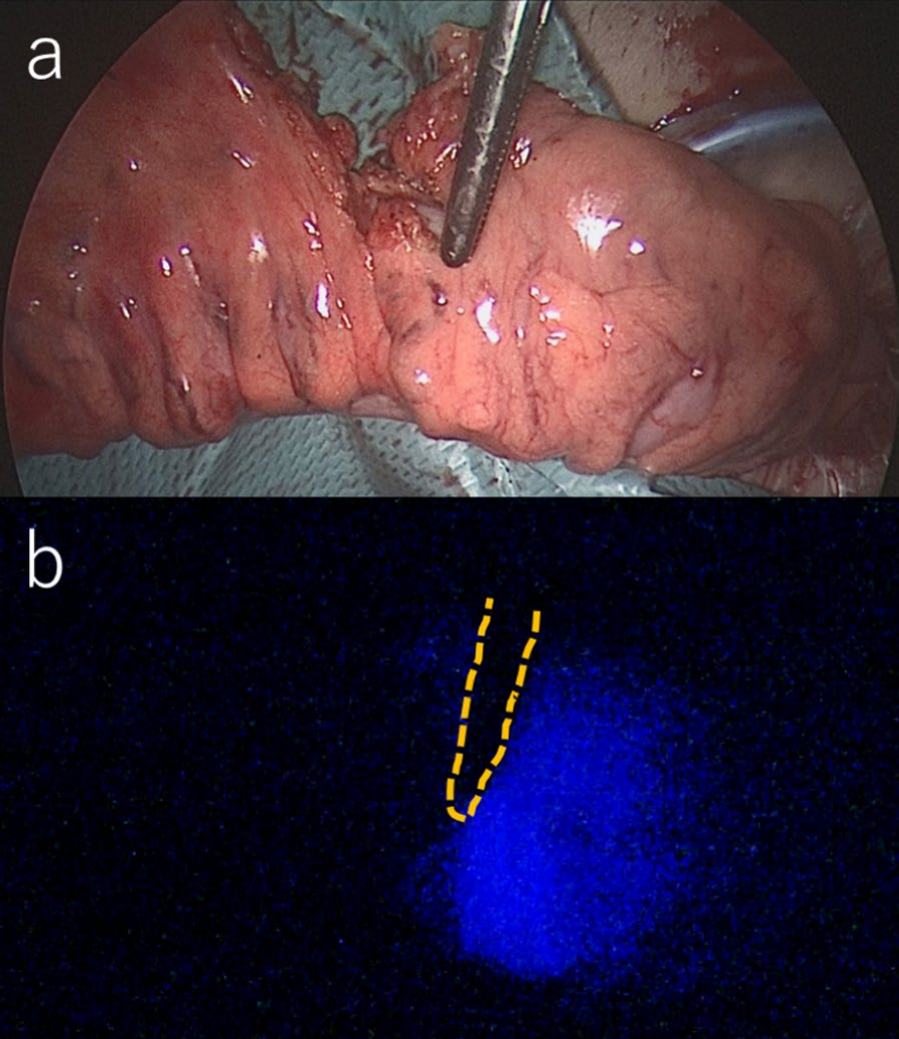
**a**, **b** After first manipulation of colonic mesentery (ICG). **a** After first manipulation of colonic mesentery. **b** After intravenous injection of ICG, the right side of the dotted line was the proximal colon, and dyed blue by ICG. The colonic segment distal to this this line was not dyed, which indicates that the proximal colon was well-perfused

**Fig. 2:**
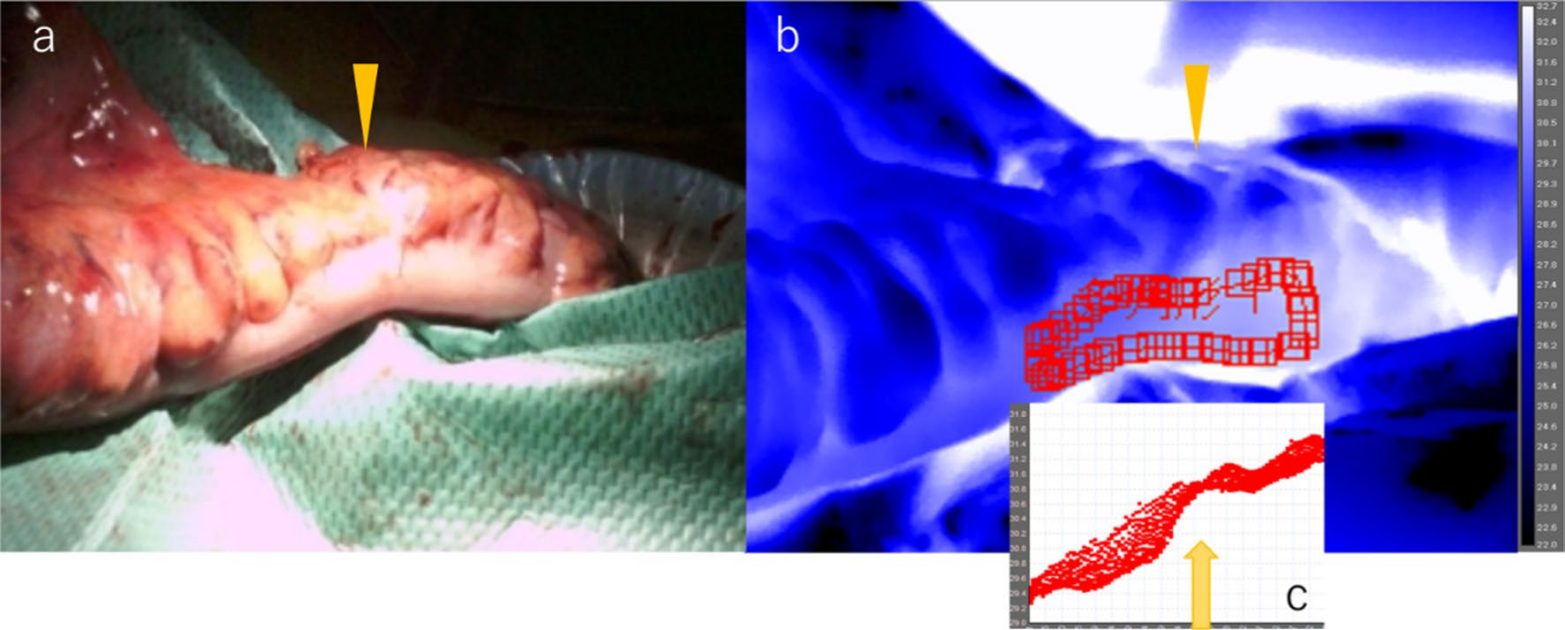
**a–c** After first manipulation of colonic mesentery (TG). **a** After first manipulation of the colonic mesentery. **b** The image when observed with TG. The right side of the arrowhead (black up-pointing triangle) was the proximal colon. **c** The temperature of the serosa. The analysis software (ResearchIRMAX) shows the zone of the temperature transition, which indicates that the temperature of the proximal colon was higher than the distal side. The arrow (↑) shows the boundary between ischemic and non-ischemic areas

Surgery was performed under general anesthesia in the lithotomy position. A laparoscopic sigmoidectomy was performed. The sigmoid colon was transected 10 cm from the anal side of the tumor, and after a small laparotomy, the sigmoid colon was extracted from the abdominal cavity. ICG (Diagnogreen; Dai-Ichi Pharm, Tokyo, Japan) was injected as a 12.5-mg bolus (0.25 mg/kg) from a peripheral vein, and ICG (Fig. 1a, b)/TG (Fig. 2a–c) was used to evaluate blood flow. We used a T530 thermal camera (FLIR Systems, Tokyo, Japan) and analysis software (ResearchIRMAX; FLIR Systems, Tokyo, Japan).

**Fig. 3:**
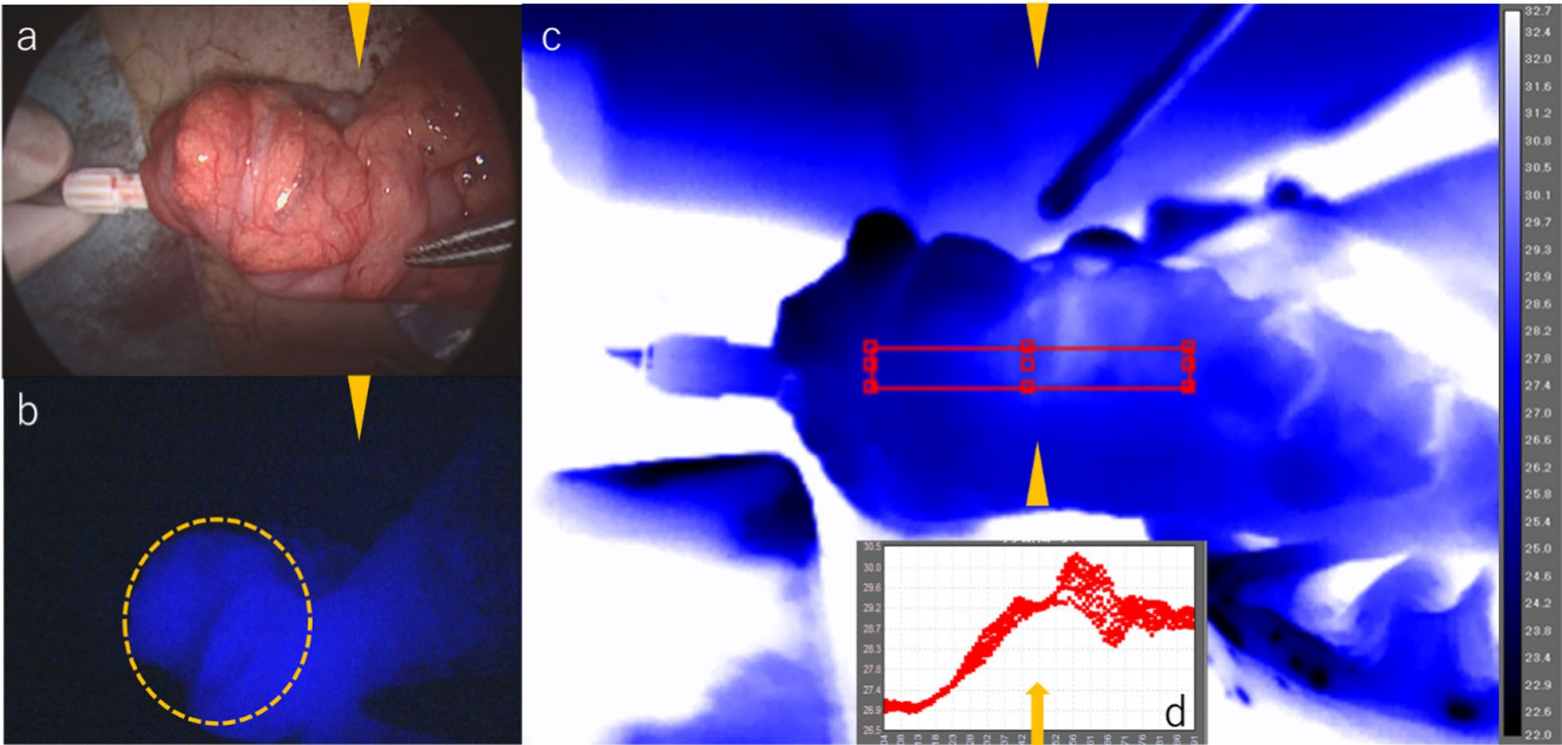
**a–d** After re-manipulation of colonic mesentery (ICG/TG). **a** The right side of the arrow head (black up-pointing triangle) was the proximal colon. **b** ICG fluorescence was observed in the region of the circle where blood flow had not been visualized. **c** The image observed with TG. The right side of the arrowhead (black up-pointing triangle) was the proximal colon. **d** The temperature of the serosa. ResearchIRMAX shows the zone of the temperature transition. This finding also means that the temperature of the proximal colon was higher than the distal side, and the arrow (↑) shows the boundary between ischemic and non-ischemic areas

After confirmation of the boundary between ischemic and non-ischemic colon tissues at the level of the mesenteric processing site, the colon was resected; however, a diverticulum was noted at the anastomotic site when the anvil head of the circular stapler was inserted and attached to the oral side of the colon when performing the anastomosis. Because there was a risk of suture failure if the anastomosis had been performed using a double stapling technique (DST), additional resection (2 cm of colon on the oral side) was needed. After further manipulation of the mesentery, ICG was administered again, and fluorescence due to the remaining ICG from the previous administration was observed in the colon that was thought to have interrupted blood flow (Fig. 3a–d). Blood flow in the colon was confirmed by TG, and a transitional zone of serosal surface temperature was observed at the mesentery processing site, which corresponded to both the tissue where blood flow had been preserved and the ischemic tissue. Therefore, additional colon resection was performed along the boundary between the ischemic and non-ischemic tissues.

After the anvil head was re-attached and the colon returned to the abdominal cavity, a DST anastomosis was performed between the oral side of the colon and the rectum. A leak test performed immediately after the anastomosis was negative, and the anastomosis appeared to have been performed successfully without any problems. A closed-suction drain was placed on the back side of the anastomotic site and the wound was closed. The postoperative course was uneventful and the patient was discharged to home 5 days after the surgery.

## Discussion

So far, the laser Doppler method, TG and ICG have been reported as the evaluation methods of intestinal blood flow. ICG fluorescence imaging is widely used in gastrointestinal surgery and is considered useful for reducing anastomotic leakage [1]. ICG is injected intravenously at concentrations ranging from 0.03 to 0.3 mg/kg. ICG binds to plasma proteins, such as lipoproteins and albumin, and is rapidly distributed throughout the body. When ICG is irradiated by light with wavelengths ranging from 750 to 810 nm, ICG emits fluorescence. Blood flow is evaluated by visualizing the fluorescence with a dedicated camera. ICG is metabolized in the liver and excreted in the bile [2]. It has been reported that ICG can significantly reduce inadequate anastomoses in rectal cancer surgery, thus ICG is widely used in Japan. The advantages of ICG fluorescent staining include the ability to objectively evaluate colonic blood flow by observing the colon through an infrared camera, a short half-life of approximately 3 min, and the ability to perform repeated examinations and record images.

The disadvantages of ICG fluorescent staining include the cost of introducing the infrared camera system and the reported cases of patients with allergies to ICG [6]. Although the half-life of ICG is short, a small amount of ICG remains in the tissues until ICG is metabolized in the liver, making it difficult to objectively judge colonic blood flow. The normal ICG 15-min retention rate, which is also used to evaluate liver function, is 0.18–0.20, and 20% remains in the body even after 15 min.

In contrast, TG evaluates colonic blood flow by identifying the zone of the temperature transition on the surface of the colonic serosa. At the site of colon mesentery surgical manipulation, the surface temperature of the serosa decreases because of ischemia. We have used TG to identify the boundary between ischemic and non-ischemic colon tissues by identifying the temperature transition zone on the surface of the colonic mesentery. Publications on reconstructive surgery have also reported that the evaluation of blood flow by TG produces results equivalent to the results produced by ICG [7–10].

The advantage of TG is that the boundary between ischemia and non-ischemia can be clearly evaluated by visualizing the surface temperature of the serosa using software. In addition, it has a relatively low cost compared to ICG and does not require the administration of drugs used for visualization. The disadvantage of TG is that the surface temperature of the intestinal mesentery can be affected by the temperature of the operating room [3]. Therefore, we have used differences between temperatures to evaluate blood flow of ischemic and non-ischemic area. We confirmed intestinal blood flow by both ICG and TG, and the boundary between ischemic and non-ischemic tissues was located similarly for both ICG and TG, suggesting that TG is not inferior to ICG for evaluating blood flow.

In this patient we first manipulated the mesentery, then objectively confirmed that each ischemic region identified by ICG and TG was similarly located; however, a diverticulum was noted at the anastomotic site, and after a second manipulation of the mesentery, blood flow was evaluated by both ICG and TG. ICG fluorescence was observed in the region where blood flow had not been visualized, as shown in Fig. 3b. We then observed that the region identified as ischemic by TG was in a different location. The reasons accounting for the differences between the locations of ischemia identified by ICG and TG are as follows: in the colon where blood flow was maintained by the first manipulation of the mesentery, ICG was taken up by the tissue and residual florescence was observed even after additional manipulation of the mesentery. In contrast, TG depicted a new zone of the temperature transition on the surface of the colon mesentery, even after additional colonic mesentery resection. Based on these observations, we decided to use the boundary between ischemic and non-ischemic colon tissues identified by TG and performed the anastomosis, which led to a successful anastomosis without complications after the additional resection.

## Conclusion

We treated a patient in whom intestinal blood flow was evaluated by TG. Because TG can identify the zone of temperature transition in the colonic serosa, TG can be used repeatedly and easily evaluates colonic blood flow. TG can be used in patients in whom additional resection is required after ICG fluoroscopy or in patients with allergies to agents used for visualization. TG is expected to enable auxiliary functions in the evaluation of colonic blood flow. Additional similar cases are needed to confirm the usefulness of TG.

## Data Availability

The data used in study are available from the corresponding author upon reasonable request.

## Abbreviations

ICG: Indocyanine green
TG: Thermography
